# Use of the Renal Artery Doppler to Identify Small for Gestational Age Fetuses at Risk for Adverse Neonatal Outcomes

**DOI:** 10.3390/jcm10091835

**Published:** 2021-04-23

**Authors:** Stephen Contag, Silvia Visentin, Katherine Goetzinger, Erich Cosmi

**Affiliations:** 1 Division of Maternal Fetal Medicine, Department of Obstetrics and Gynecology and Women’s Health, University of Minnesota, Minneapolis, MN 55455, USA; scontag@umn.edu; 2 Department of Women and Child Heath, University of Padua School of Medicine, 35122 Padova, Italy; silvia.visentin.1@unipd.it; 3 Division of Maternal Fetal Medicine, Department of Obstetrics, Gynecology & Reproductive Sciences, University of Maryland School of Medicine, Baltimore, MD 21201, USA; Goetzingerk@maryland.edu

**Keywords:** renal artery, Doppler, pulsatility index, cerebral–placental ratio

## Abstract

Objective: To measure the sensitivity and positive predictive value (PPV) for an adverse neonatal outcome among growth-restricted fetuses (FGR) comparing the cerebral–placental ratio (CPR) with the cerebral–renal ratio (CRR). Methods: Retrospective analysis of 92 women who underwent prenatal ultrasound at the University of Maryland and the University of Padua. Renal, middle cerebral and umbilical artery Doppler waveforms were recorded for all scans during the third trimester. The last scan prior to delivery was included for analysis. We calculated the test characteristics of the pulsatility indices (PI) of the umbilical and renal arteries in addition to the derived CPR and CRR to detect a composite adverse neonatal outcome. Results: The test characteristics of the four Doppler ratios to detect increased risk for the composite neonatal outcome demonstrated that the umbilical artery pulsatility index had the best test performance (sensitivity 64% (95% CI: 47–82%), PPV 24% (95% CI: 21–27), and positive likelihood ratio 2.7 (95% CI: 1.4–5.2)). There was no benefit to using the CRR compared with the CPR. The agreement between tests was moderate to poor (Kappa value CPR compared with CRR: 0.5 (95%CI 0.4–0.70), renal artery PI:−0.1 (95% CI −0.2–0.0), umbilical artery PI: 0.5 (95% CI 0.4–0.7)). Only the umbilical artery had an area under the receiver operating curve that was significantly better compared with the CPR as a reference (*p*-value < 0.01). Conclusions: The data that we present do not support the use of renal artery Doppler as a useful clinical test to identify a fetus at risk for an adverse neonatal outcome. Within the various indices applied to this population, umbilical artery Doppler performed the best in identifying the fetuses at risk for an adverse perinatal outcome.

## 1. Introduction

Studies regarding fetal adaptation to decreased placental perfusion, nutrient transfer or gas exchange in the fetal sheep model have reported that blood is shunted away from less essential organs such as the kidney, bowel and musculoskeletal system, in order to conserve blood flow to the adrenal glands, heart and brain [1,2,3,4,5]. This brain-sparing effect results from a decreased impedance to flow in the brain and heart, with a concurrent increase in resistance to flow within the peripheral arterial system. This is presumed to be mediated by the sympathetic nervous system [6], and the presumed mechanism is hypoxemia, which increases catecholamine concentration in fetal sheep secondary to the peripheral fetal chemoreflex response [2,5,6]. Relative hypoxia leads to increasing beta-adrenergic activity, fetal heart rate and blood pressure. Alpha-adrenergic activity increases later in gestation and does not have a marked effect on blood pressure [7]. As pregnancy advances, catecholamine secretion can be achieved by progressively milder degrees of hypoxemia. These medullary and peripheral cardiovascular responses are critical to circulatory adjustments in response to hypoxemia [2,5,6].

Ultrasound using spectral Doppler waveforms, originally reported for the umbilical artery, demonstrated that a decrease in the diastolic to systolic ratio identifies growth-restricted fetuses at risk for adverse outcomes secondary to hypoxemia [8]. Histopathological studies confirmed a correlation between vascular resistance in the umbilical arteries and placental vascular tree damage [9].

Among fetuses with early-onset growth restriction at <32 weeks, increasing resistance in the umbilical artery (UA) is most efficient at identifying fetuses at risk for adverse neonatal outcomes, with the severity of the increase and gestational age used to decide on proceeding with delivery [10]. Maternal hypertensive disease, abnormal fetal heart rate (FHR) testing or evidence of increasing resistance to flow in the fetal ductus venosus are usual indications for delivery [10]. The increased UA resistance patterns have been shown to develop weeks, if not months, before late-onset decelerations of the FHR are observed [1].

Fetuses diagnosed as being growth-restricted after 32 weeks are reported to have increased perinatal mortality in the absence of increased resistance in the UA [11,12]. Use of the UA to evaluate a fetus with FGR has resulted in false negatives, leading to use of the cerebral–placental ratio (CPR) as a more sensitive test [13,14]. Currently, surveillance for fetuses with FGR at greater than 32 weeks includes measurement of the UA resistance indices or the CPR. The CPR is reported to have greater specificity for the prediction of FGR fetuses at risk for adverse outcomes, with a positive test associated with odds ratios of greater than 10 [13,15]. Its use has also been proposed for surveillance of appropriately grown fetuses [16]. Based on these observations, recommendations for the surveillance of a fetus with FGR include both umbilical and middle cerebral artery (MCA) spectral Doppler waveform assessments using the CPR when greater than 32 weeks gestational age [17]. The CPR is reported to improve the prediction of adverse neonatal outcomes in fetuses with FGR [14,15,16,18,19].

Although the CPR has been described as a better test to assess risk for adverse outcomes in the third trimester, the positive predictive value (PPV) for the test across various populations is not consistent and use of the CPR compared with the UA resistance indices to identify the fetus with FGR at risk is not universally accepted [20].

The MCA pulsatility index (MCAPI) by itself has limited predictive accuracy in determining the risk for a compromised neonatal outcome [21]. The fetal adaptive response underlying the use of the CPR is a decrease in the MCAPI in the setting of a stable UA pulsatility index (UAPI), which suggests cerebral vasodilation secondary to relative hypoxia, without increased UAPI. This is presumed to occur secondary to poor placental exchange, and not necessarily due to placental vascular disease or poor perfusion [16,22]. Having a consistently suppressed CPR has been shown to have the highest predictive value for adverse outcome, particularly after 37 weeks [18,23].

Based on this precept, it would be important to establish whether evaluation of the renal artery (RA) pulsatility index (RAPI) and the derived cerebral–renal ratio (CRR) improves the detection of centralization of flow in the third trimester, compared with use of the UAPI and the CPR. Our primary objective is to measure the sensitivity and PPV for an adverse neonatal outcome among two different populations of FGR fetuses applying both the CPR and the CRR. Our hypothesis is that the test characteristics of a CRR among different populations of pregnancies affected by FGR may provide increased test accuracy compared with the CPR.

## 2. Methods

This is a retrospective analysis of data used for clinical management. It was approved by the University of Maryland Institutional Review Board (authorization HP-00072884) and by the Ethics Committee of the University of Padua (authorization P1826). Women who underwent prenatal ultrasound at the Center for Advanced Fetal Care at the University of Maryland Medical Center (UMMC) and at the University of Padua (UP) between 1 June and 1 December 2016 underwent assessment of the UAPI, MCAPI and the RAPI. Prior to this time, each center routinely evaluated the uterine, umbilical and middle cerebral artery Doppler waveforms and, in May 2016, after deciding to incorporate the renal artery into our fetal surveillance algorithm, began to obtain renal artery spectral Doppler information at both centers, regardless of the indication for the ultrasound. The last scan prior to delivery was included for analysis.

The study population included women whose fetus was identified as having an estimated fetal weight <10th percentile for gestational age and were identified from the corresponding electronic database at each medical center. We included women with singleton pregnancies who underwent growth scans during a 6-month period. We calculated gestational age from the last menstrual period if it was consistent with the crown rump length (CRL) obtained at first trimester nuchal translucency scan. If there was greater than 7-day discordance, we used gestational age calculated from the first trimester CRL [24].

Renal, middle cerebral and umbilical artery Doppler assessments were recorded as a component of all prenatal ultrasounds during the third trimester. Women underwent testing for various clinical indications including a history of stillbirth, FGR or preeclampsia; or a diagnosis of pregestational diabetes, gestational diabetes, chronic hypertension or gestational hypertension.

We excluded all cases with a known fetal structural abnormality, genetic syndrome or aneuploidy. We also excluded pregnancies where neonatal outcomes were not available. Fetal biometry was performed according to a standard protocol at each institution and the fetal weight calculated [25]. The percentile distribution provided by the proprietary software in the ultrasound reporting system at each site was applied. A standardized technique was applied by qualified ultrasound technicians to obtain all MCA and UA spectral Doppler measurements [26]. The first month of data collection served to standardize the technique for RA Doppler assessment and the quality of the measurements was reviewed throughout the 6-month period for consistency. The RA Doppler signals were measured with an angle of insonation of less than 30°, in a coronal plane, with placement of the Doppler gate 1–2 mm from the origin of the renal artery at the descending aorta. The RA closest to the probe was chosen if both were easily found; otherwise, the side with the best signal was recorded. Two waveform segments that completely filled the width of the screen were recorded. The two waves with the best image in each segment were used for manual trace and the averaged data set was saved. We abstracted the spectral Doppler values for the MCAPI, UAPI and RAPI. The CPR (MCAPI/UAPI) and the CRR (MCAPI/RAPI) were calculated from the three measured values [27].

We plotted the measured and derived values for the UA, MCA and CPR on published reference curves [28,29]. The RAPI and CPR reference curves were derived from a healthy population of adequately grown fetuses [30]. We used a value of less than the 5th percentile to identify abnormal values for the CPR and the CRR, and greater than the 95th percentile for the UAPI and the RAPI. We did not include the MCAPI for this analysis as it has been shown not to be an accurate test for prediction of adverse outcomes [21].

Maternal descriptive and neonatal outcome information was abstracted from electronic medical records. Maternal information included past medical history, pregnancy history, age and race. Neonatal information included gestational age at birth, newborn birthweight, Apgar scores, mode of delivery and sex. A composite adverse neonatal outcome included Apgar score <7 at 5 min, neonatal intensive care unit (NICU) admission for more than 48 h, assisted ventilation for more than 6 h after delivery, fetal metabolic acidosis (arterial cord blood pH less than 7.2 and base excess >12), fetal or neonatal death and intraventricular hemorrhage (IVH) Papille Grade III or IV.

### Statistical Analysis

We compared the maternal and neonatal baseline characteristics using the chi-square test or Fisher’s exact test for categorical variables and the Kruskall–Wallis test for continuous variables. Our primary outcome was the proportion of growth-restricted fetuses with a CRR <5th percentile compared with the proportion of fetuses with CPR <5th percentile who developed the composite neonatal outcome. The secondary outcomes consisted of comparing the test characteristics for the CPR, CRR, UAPI and RAPI >95th percentile, to predict the subsequent development of any component of the composite neonatal outcome. The test characteristics for each one of the Doppler parameters were calculated. These included test sensitivity, specificity, false positive and false negative rates, positive and negative predictive values and the positive and negative likelihood ratios. The CPR was compared against the CRR, RAPI and the UAPI using the area under the receiver operating characteristics curve (AUROC). A *p*-value < 0.05 was considered significant. All analysis was carried out using SAS 9.3 (SAS, Cary, NC, USA).

## 3. Results

At UMMC, there were 2852 women who underwent ultrasound assessments, of which 2289 were excluded for missing outcome data, leaving 563 women with complete data, of which 44 had a diagnosis of FGR. At the UP, there were 750 ultrasounds performed among 600 women, of which 48 had a diagnosis of FGR with complete outcome data. In total, there were 92 women with a fetus diagnosed with an estimated fetal weight <10th percentile who had complete Doppler and neonatal outcome data available for analysis. Descriptive information regarding the women, the deliveries and their infant outcomes are shown in Table 1 and Table 2. Some infants developed more than one complication.

The mean and median values for each of the Doppler parameters measured are shown in Table 3 with a statistically significant difference in the mean value obtained for the RAPI (2.29, (SD 0.42) versus 2.07, (SD 0.50), *p*-value 0.04)) at Maryland and Padua, respectively. There were no differences for any of the other indices.

**Figure 1:**
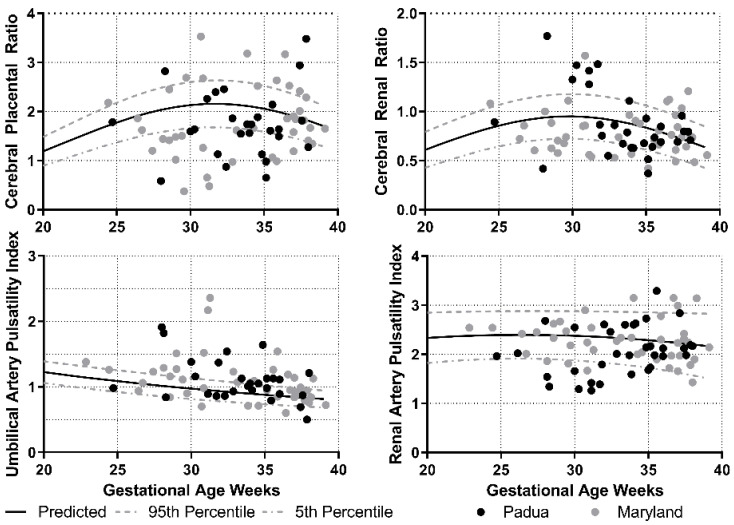
Doppler parameters and distribution by medical center.

**Figure 2:**
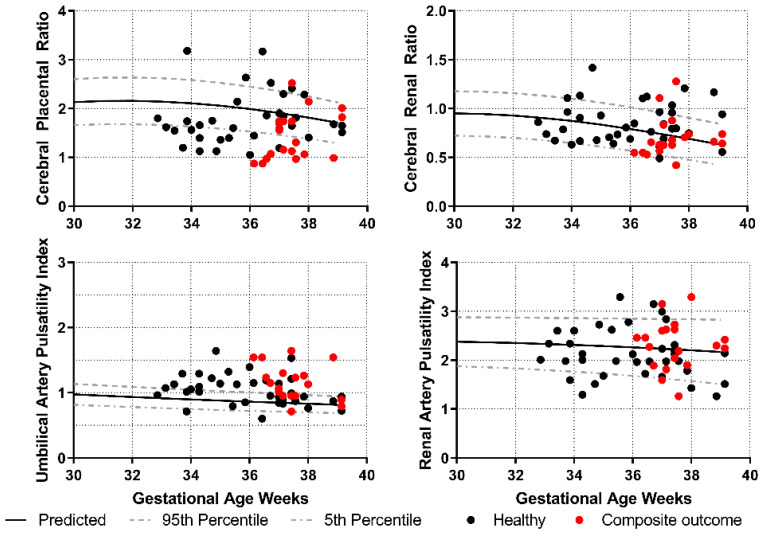
Doppler parameters and distribution by neonatal outcome.

We plotted the values for the measurements obtained from both Maryland and Padua against the reference curves and observed a visually similar distribution between both groups (Figure 1). We also plotted the most recent value prior to delivery for all fetuses born at or after 32 weeks (when these tests were most commonly obtained and clinically relevant) according to reported neonatal outcomes (Figure 2). It is apparent that a larger proportion of FGR fetuses lie below the 5th percentile of the CPR compared with the CRR. The test characteristics of the four Doppler parameters to detect increased risk for the composite neonatal outcome demonstrated that the UAPI had the best performance. Agreement between tests was moderate to poor (Table 4).

**Figure 3:**
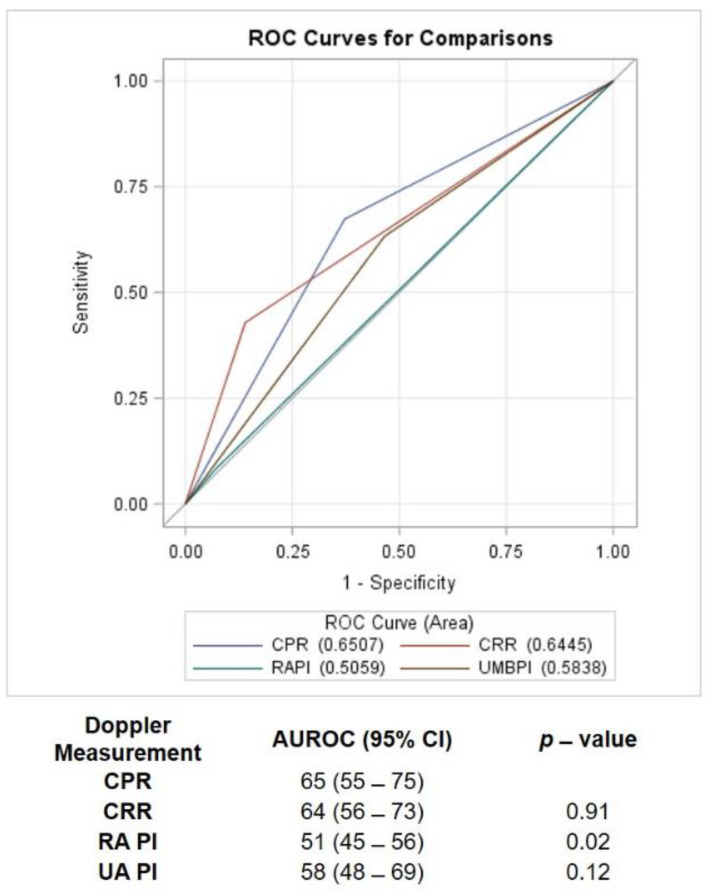
Comparison of the area under the receiver operating characteristics curve (AUROC) for each test to detect the composite neonatal outcome. ROC: receiver operating characteristics, PI: pulsatility index, CPR: cerebral–placental ratio, CRR: cerebral–renal ratio.

The AUC of each test to detect the primary outcome and the comparison between tests is shown in Figure 3. When we compared the AUROC using the Mann–Whitney test for receiver operating curve contrasting estimations, we only detected a significant difference for the RAPI, which was significantly less efficient compared with the CPR. The other three indices performed similarly to each other.

## 4. Discussion

The RAPI or the CRR did not improve the detection of FGR pregnancies at risk for adverse outcomes, compared with the currently recommended CPR or UAPI. The distribution of all the measured and derived values from both institutions did not show any visually apparent difference between the two sites, despite a statistically significant difference in the mean RAPI value. This suggests that despite being statistically different, this was not clinically relevant.

Our results show that among pregnancies complicated by FGR, fetal RA resistance does not increase among fetuses with FGR who develop short-term adverse outcomes. Our data are consistent with the absence of significant changes in the impedance to flow within the renal system, which is thought to occur because of relative fetal hypoxia among some FGR fetuses [3,6,15,31]. This is contrary to what we had expected to observe based on conventional views of the fetal response to acute intrapartum hypoxia [5]. Although we did not assess partial pressures of oxygen or oxygen saturation within the fetal blood, indirect signs of fetal placental vasculopathy are often evidenced by an increase in the UAPI, considered a sign of placental vascular disease [9,32,33]. If the relative chronic hypoxia in the FGR fetus is not a sign of hypoxia and vasculopathy but, rather, is secondary to poor or limited diffusion in the placenta, the expected decrease in CPR would be due to decreased impedance in the cerebral vasculature [34,35].

In this study, we found poor agreement between the CPR and the CRR among fetuses who developed any component of the composite neonatal outcome. The RAPI had the lowest agreement with the CPR, suggesting that the hemodynamic changes described above, and consistent with a decreasing CPR, do not appear to increase renal arterial impedance to flow. One mechanism for these findings may be from studies demonstrating that chronically hypoxic sheep fetuses gradually lose their renal and femoral vasoconstrictive response to hypoxia [36]. There is also evidence that the vasoconstrictive response may not be fully active until the fetus is at term [5,37]. Adrenergic stimulation of the fetus is shown to cause hypertension and a decrease in heart rate and in renal, visceral, upper and lower extremity blood flow as early as mid-gestation [38,39]. This vasoconstrictive response to norepinephrine progressively increases throughout pregnancy, continuing after birth, when responses to adrenergic stimulation may be more effective [40].

Prior work by Galan et al. had suggested that the brain-sparing effect results from peripheral sympathetic activation of the vascular system with increased peripheral systemic resistance and passive redistribution of blood flow to the brain [3,6,31,40]. This passive redistribution is presumed to be associated with peripheral fetal hypertension [41]. More recent work in preterm newborns has demonstrated that cerebral blood flow is passive prior to 24 weeks and gradually becomes evident from 24 to 32 weeks. After 32 weeks, cerebral systolic blood flow velocity is autoregulated in response to changes in mean arterial pressure; however, diastolic blood flow continues to be passive to changes in systemic arterial pressure and blood volume [42]. The fetal responses to acute hypoxic events follow well these well-defined patterns of peripheral vasoconstriction, but do not apply to a chronically affected fetus with growth restriction, in which these responses appear to be blunted [36].

This is consistent with an increase in the pulsatility index in the aortic isthmus of FGR fetuses, which appears to reflect decreasing diastolic flow secondary to passive redistribution of blood redirected to the cerebral vasculature [43]. Most of the fetal aortic blood flow is provided by the right ventricle, with only thirty percent of flow provided through the aortic arch [44]. Increasing impedance to flow in the descending aorta is transmitted mostly to the right ventricle, with increased flow across the foramen ovale to the left atrium, ventricle and the aorta [45,46]. An acute hypoxic event in the fetus with vasoconstriction and elevated blood pressure has been shown to affect renal arterial flow secondary to decreased flow into the abdominal aorta; however, this mechanism has not been observed in cases of chronic hypoxia associated with FGR [36,47,48,49].

Previous studies have shown that the CPR is more specific but less sensitive than the UA resistance indices to identify fetuses at risk for adverse neonatal outcomes after 32 weeks [13,17,36,47,48,49]. Our results demonstrated that the CPR had the highest sensitivity, and, although the RAPI and the CRR had the highest specificity, they also had the lowest test sensitivity. In terms of overall test performance, the CPR appeared to have the highest value, but this was not significantly different from that of the CRR or the UAPI. Only the RAPI performed significantly less efficiently than the CPR due to its low sensitivity.

There are several published reports on the use of the renal artery for the evaluation of late-onset FGR fetuses [50,51]. An increase in the RAPI has been reported to occur with FGR complicated with oligohydramnios, but not among normally grown fetuses [52,53]. The observed changes in RAPI among FGR fetuses have been limited and not always consistent across populations, with two reports of no change [11,51] and one other reporting increasing RAPI, which correlates with fetal hypoxia and acidosis [54].

The evidence provided by this study did not support the use of the RA for surveillance of the FGR fetus to provide risk assessment for an adverse neonatal outcome. Two mechanisms appear to limit the utility of the RAPI and CRR. The first is that, although the peripheral circulation, including the RA, has been observed to constrict in response to acute neurohumoral stimuli secondary to hypoxia and acidosis, in our FGR cohort, we found no such effect. This is consistent with FGR fetuses following a pattern of chronic hypoxia that is less sensitive to a humoral vasoconstrictive response [37]. A second factor is that RA spectral Doppler studies have consistently reported decreasing resistance with increasing gestational age [30,55,56]. This could decrease the impact of a lower MCAPI. Although this effect is also observed with the CPR, the wider reference range for the RA relative to the UA may dampen the variations in the CRR compared with the CPR. A third factor is the relatively high resistance observed in the RA compared with the UA [30]. The higher resistance would also lead to overall lower values for the CRR, requiring even higher elevations of the RAPI to decrease the CRR outside of the observed reference ranges.

The main limitation to this study is the number of cases that are included. It is also possible that the results that we have obtained will not necessarily apply to other populations of fetuses that are found to have FGR due to the multiple causes of this condition. A second limitation is the lack of direct assessment of fetal hypoxia. It is unlikely that we would be able to obtain such information as it would require some form of invasive testing. A strength of this study is that we standardized the assessment of the RA measurements within and across the two institutions. This provided adequate and reliable Doppler waveform patterns for analysis. The two study cohorts were collected concurrently, which would decrease the effect of temporal changes in perinatal care on neonatal outcomes. Although the data were collected in 2016, we feel that the methodology utilized for data collection and analysis, as well as the technology used for Doppler assessments, has not varied substantially over the past 5 years and would not affect the results. The data presented support published work suggesting that centralization of flow in the chronically hypoxic fetus is not an adrenergic vasoconstrictive response within the RA, but, rather, it may result from increased impedance to flow in the placental vasculature with passive redirection of flow to the brain.

Spectral Doppler waveform analysis has become an important modality to identify the FGR fetus at increased risk for perinatal morbidity and mortality. The data that we present do not support the use of the RAPI or CRR as a useful clinical test to identify a fetus at risk or to determine the timing of delivery. Within the various indices applied to this population, UAPI performed the best in identifying those fetuses that subsequently developed any significant adverse perinatal outcome. The finding that the RAPI did not increase also adds to our understanding of the physiological adaptation of the fetus to growth restriction.

## Figures and Tables

**Table 1:** Maternal characteristics.

	All (92)
Variable	Median (range)
Gravida	2 (1–3)
Para	1 (0–2)
BMI	30.5 (23.3–37.3)
EFW	1932 (1275–2183)
Percentile	7 (4–9)
AFI	13.1 (11.1–16.7)
Gestational age at scan	34 (32–36)
Scan to delivery time	2.14 (0.9–4.0)
	Number (%)
Preeclampsia	15 (16)
Chronic hypertension	6 (6.5)
Gestational diabetes	7 (7.6)

BMI: body mass index, EFW: estimated fetal weight, AFI: amniotic fluid index.

**Table 2:** Delivery and neonatal outcomes ^1^.

	Total (92)
Variable	Median (range)
Gestational age at birth	37 (34–39)
Birthweight	2305 (1630–2660)
NICU length of stay	4 (0–25)
	Number (%)
Mode of delivery	
Vaginal	35 (39)
Cesarean	52 (58)
Operative vaginal	2 (2)
Unknown	3 (2)
Indication for delivery: non-reassuring fetal status	27 (30)
Neonatal outcomes	
Apgar <7 at 5 min	7 (8)
Umbilical artery pH <7.2	24 (26)
Umbilical artery BE >8	19 (21)
Abnormal pH and BE ^2^	19 (21)
NICU admission >48 h	37 (40)
Neonatal ventilation >6 h	9 (10)
Neonatal complication ^3^	49 (53)

^1^ There were no cases of neonatal necrotizing enterocolitis, seizures, intraventricular hemorrhage grade III-IV, hypoxic ischemic encephalopathy or death. ^2^ Abnormal pH and BE: presence of both pH less than 7.2 and BE (base excess) greater than 12. ^3^ Neonatal complication includes: Apgar less than 7 at 5 min, abnormal pH and BE, requiring assisted ventilation for more than 6 h and requiring admission to NICU for more than 48 h. BMI: body mass index, EFW: estimated fetal weight, AFI: amniotic fluid index, BE: Base Excess; NICU: neonatal intensive care unit.

**Table 3:** Comparison of Doppler parameters for each site.

	Maryland	Padua	
Test	Mean (SD)	Mean (SD)	*p*-Values
Umbilical artery pulsatility index	1.15 (0.52	1.10 (0.33)	0.6
Renal artery pulsatility index	2.29 (0.42)	2.07 (0.50)	0.04
Cerebral–placental ratio	1.76 (0.72)	1.74 (0.68)	0.93
Cerebral–renal ratio	0.79 (0.23)	0.88 (0.34)	0.24

**Table 4:** Test characteristics for composite neonatal outcome and 95% confidence intervals.

	CPR	CRR	Renal PI	Umbilical PI
Sensitivity (%)	67 (54–80)	43 (29–57)	8 (2–20)	63 (50–77)
Specificity (%)	63 (48–77)	86 (76–96)	93 (81–99)	54 (39–68)
False positive rate (%)	37 (23–52)	14 (5–28)	7 (2–19)	47 (32–61)
False negative rate (%)	33 (20–46)	57 (43–71)	92 (80–98)	37 (23–50)
Positive predictive value (%)	67 (54–80)	78 (62–93)	57 (18–90)	61 (47–74)
Negative predictive value (%)	63 (48–77)	57 (45–69)	47 (37–58)	56 (41–71)
Positive likelihood ratio	1.8 (1.2–2.8)	1.8 (1.3–2.5)	1.1 (0.6–2.1)	1.4 (0.9–2.1)
Negative likelihood ratio	0.6 (0.4–0.9)	0.6 (0.4–0.8)	0.9 (0.5–1.8)	0.7 (0.5–1.1)
McNemar’s *p*-value	Reference	<0.01	<0.01	0.9
Cohen’s kappa value	Reference	0.6 (0.5–0.7)	−0.04 (−0.1–0.03)	0.5 (0.3–0.6)

PI: pulsatility index, CPR: cerebral–placental ratio, CRR: cerebral–renal ratio.

## Data Availability

3rd Party Data. Restrictions apply to the availability of these data. Data was obtained with permission from the University of Maryland and the University of Padua, and are available from the authors with the permission of both academic centres.
